# Design and applications of stretchable and self-healable conductors for soft electronics

**DOI:** 10.1186/s40580-019-0195-0

**Published:** 2019-08-01

**Authors:** Yue Zhao, Aeree Kim, Guanxiang Wan, Benjamin C. K. Tee

**Affiliations:** 1 NUS Graduate School for Integrative Sciences and Engineering, National University of Singapore, Singapore, 117456 Singapore; 2 Department of Material Science and Engineering, National University of Singapore, Singapore, 117575 Singapore; 3 Department of Electrical and Computer Engineering, National University of Singapore, Singapore, 117583 Singapore; 4 Institute for Health Innovation and Technology, (iHealthtech), National University of Singapore, Singapore, 117599 Singapore; 5 Singapore Institute of Manufacturing Technology, Agency for Science, Technology and Research (A*STAR), Singapore, 138634 Singapore

**Keywords:** Stretchable electronics, Soft material, Stretchable conductors, Elastic conductor, Self-healing, Nanocomposite, Electronic skin

## Abstract

Soft and conformable electronics are emerging rapidly and is envisioned as the future of next-generation electronic devices where devices can be readily deployed in various environments, such as on-body, on-skin or as a biomedical implant. Modern day electronics require electrical conductors as the fundamental building block for stretchable electronic devices and systems. In this review, we will study the various strategies and methods of designing and fabricating materials which are conductive, stretchable and self-healable, and explore relevant applications such as flexible and stretchable sensors, electrodes and energy harvesters.

## Introduction

Stretchable electronic devices have received increasing attention by researchers globally as they have the potential to be applied in many innovative fields such as epidermal electronic devices [1, 2], biomedical engineering [3, 4], healthcare monitoring [5–8], soft robotics [9–12], electronic skins [13–15] and human–machine interfaces [16]. Based on the tremendous growth of nanomaterials and nanofabrication technologies during the past decades, development of stretchable electronics has achieved remarkable progress, and they are considered as next-generation electronic devices that can augment traditional rigid silicon-based electronic devices for interfacing with the human skin or on curved, deformable interfaces. Broadly, stretchable electronics are devices that consist of electronic materials and/or circuits integrated onto stretchable substrates. Compared with rigid printed circuit boards, stretchable electronic circuits have the ability to mechanically bend, twist, compress and stretch as a result of using elastomeric soft substrate materials.

**Fig. 1:**
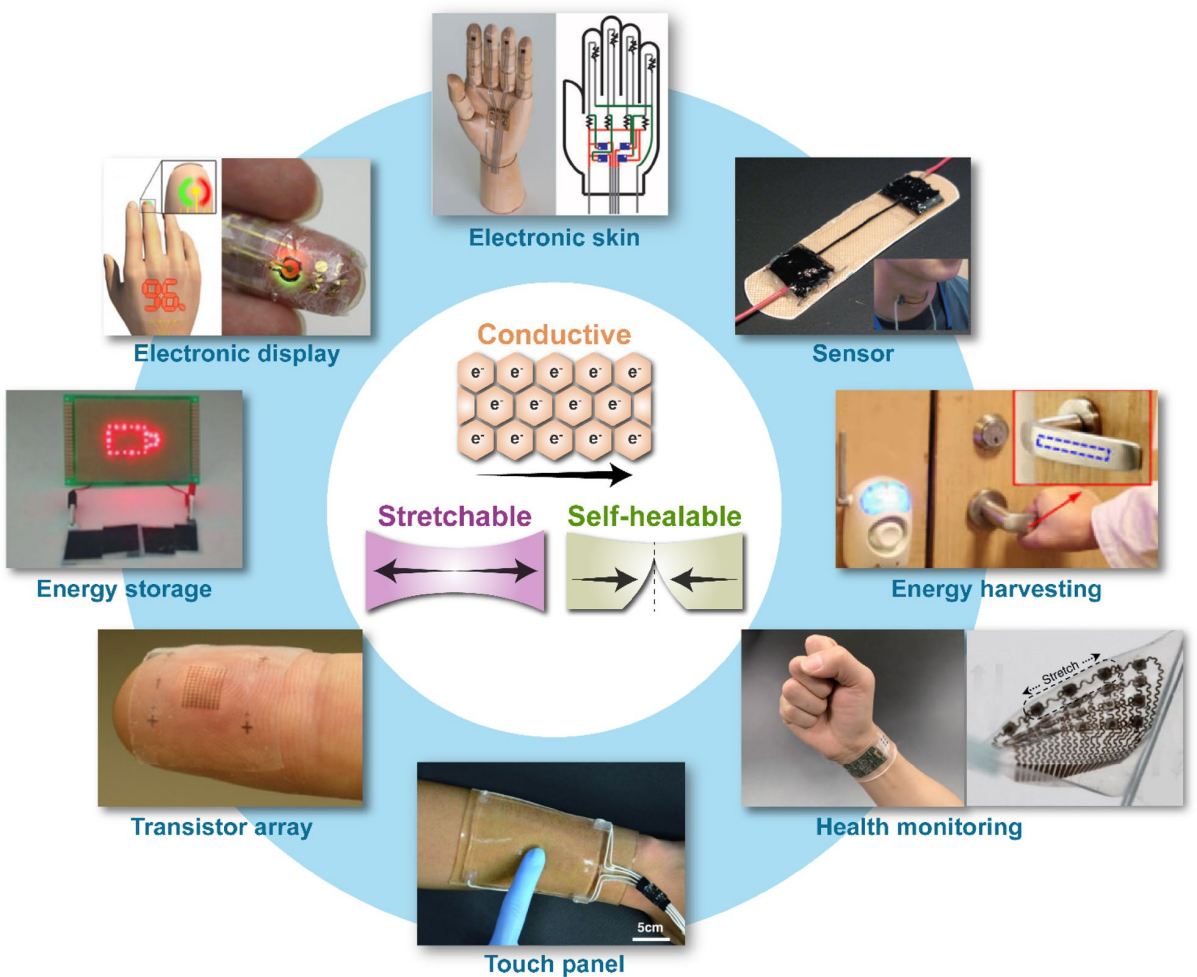
Illustration and potential applications of stretchable and self-healable conductors. Figures sequence is from top and clockwise. Illustrated figure of an electronic skin. Reproduced with permission [15]. Copyright 2015, American Association for the Advancement of Science. Photograph of a stretchable strain sensor. Reproduced with permission [17]. Copyright 2011, Nature Publishing Group. Photograph of an energy harvesting device. Reproduced with permission [18]. Copyright 2014, American Chemical Society. Photograph (left) of a health monitoring wearable sensor array. Reproduced with permission [21]. Copyright 2016, Nature Publishing Group. Photograph (right) of a stretchable ultrasonic device for blood pressure waveform monitoring. Reproduced with permission [22]. Copyright 2018, Nature Publishing Group. Illustrated figure of a highly stretchable and transparent ionic touch panel. Reproduced with permission [23]. Copyright 2016, American Association for the Advancement of Science. Photograph of a stretchable transistor array on fingertip [19]. Reproduced with permission. Copyright 2018, Macmillan Publishers Limited. Photograph of healed zinc ion battery. Reproduced with permission [24]. Copyright 2019, Wiley–VCH. Photograph of an electronic display. Reproduced with permission [20]. Copyright 2016, American Association for the Advancement of Science

For electronic functionality, electrical conductors that can stretch are the critical building blocks in potential electronic applications such as artificial electronic skins [15], smart sensors [17], energy harvester [18], transistor array [19], light-emitting diode (LED) display [20], health monitoring [21, 22], touch panel [23] and energy storage devices [24] (Fig. 1). Based on the functionalities of these new generation devices, stretchable conductors require the ability to withstand high mechanical strains (> 50%) and high electrical conductivity. The understanding of nanoscale mechanics, material properties and structure–property relationships, in combination with micro-fabrication and material processing techniques, have helped realize various forms of stretchable conductors. In addition, everyday usage of the device: stretching, twisting, impact and temperature fluctuations can all lead to damage, and the damage may not always be visible or accessible. This will negatively impact the performance and lifespan of the stretchable devices. Thus, the ability to automatically repair the damage, much like the ability of human skin to self-heal, is another desirable property of the next generation electronic devices.

Materials play an important role in developing stretchable conductors to achieve the desired mechanical and electrical properties. To achieve high stretchability for applications, elastomeric materials, such as polydimethylsiloxane (PDMS) [25], Ecoflex [26] and polyurethane (PU) [27], are usually used as the substrate or matrix to integrate with other parts of the system. On the other hand, to achieve high conductivity, traditional metallic conductors, or conductive nanomaterials such as metallic nanowires [28] and carbon nanotubes (CNT) [29] which are dispersed in the matrix, work as conductive fillers to increase conductivity. Numerous works in the literature have studied the interactive behaviors between various conductive fillers and matrixes which have different mechanical properties [29–33]. Integration of the two different phases, which typically has very different mechanical properties, presents a significant challenge, and at the same time, excellent opportunities for researchers to tackle. To-date, various solutions have been developed, with each of them utilizing unique and creative techniques such as coating [34, 35] and patterning [36–39].

The strategies of fabricating stretchable conductors can be classified broadly into three types: (i) geometric engineering of non-stretchable components [5, 40–43], (ii) intrinsically stretchable material development [29, 33, 34, 44], and (iii) combination of first two types [45–49]. Table 1 shows a general comparison between the first two approaches of fabricating stretchable electronics based on the methods of mechanically geometric design and material synthesis respectively.

**Table 1:** Comparison between two approaches fabricating stretchable electronics based on ways of mechanically geometric design and material synthesis

	Fabrication complexity	Conductivity and stretchability	Integration and stability	Stretching direction
Intrinsic material synthesis	Simple, easy handling	Low conductivity and stretchability	High integrity	Multi-axial
Structural design	Complicated, hard handling	High conductivity and stretchability	Low integrity	Single direction

One of the first strategy to produce stretchable conductors is to utilize intrinsic conductive material synthesis. The most commonly used method to achieve this purpose is to disperse nanomaterial fillers into the elastomeric matrix, combining advantages of electrical conductivity of the nanomaterial fillers and mechanical stretchability of the matrix material [50, 51]. To achieve high performance in both mechanical and electrical behaviors, a balance needs to be maintained between stretchability and conductivity. This is because more fillers incorporated into the elastomeric substrate will increase electrical conductivity while causing the whole composite material to be stiffer [27, 52]. In addition, most elastomeric materials used to form the stretchable components are solution-based, and solution processing of multi-stacked layers in the functional devices must address problems associated with dissolution, mixing, or cracking of the underlying elastomeric layer. Therefore, methods of adding fillers and their dispersion process, as well as adhesive bonding chemicals like surfactants, play critical roles in improving the quality of the whole composite [50].

Another strategy uses geometric design of non-stretchable conductive material within the elastic matrix, which enables the composite to have the ability to stretch. Stretchable electronics can be created by a combination of rigid electronic islands and stretchable interconnects [53, 54]. Generally, composites produced by this method have high stretchability and remain stable under applied deformation. However, it has several disadvantages: the fabrication methods require multiple steps, have relatively high costs, are hard to control and it is difficult to achieve scalable manufacturing. Also, there are usually issues with integrating the stretchable interconnects and the matrix. Thus, solving these problems are critical challenges in this mechanical method. Since both strategies have their own advantages and limitations as shown in Table 1, researchers are exploring combinations of these two methods to achieve better performance in stretchable conductors.

Similarly, to achieve self-healing, different strategies can be adopted. In extrinsic self-healing systems, healing agents, usually monomers, can be encapsulated and dispersed within the matrix. When cracks rupture the material and hence the encapsulation, the agents flow out, polymerize, and heal the damage. Alternatively, intrinsically self-healing materials based on supramolecular interactions, like hydrogen bonds or Diels–Alder reactions, can be designed. Self-healing via encapsulated healing agents can occur without intervention at the damage site but face the challenge of repeatable healing as the healing agent is depleted. Attempts have been made to overcome this disadvantage, mainly by microvascular design, where a network allows healing agents to flow to that site. Intrinsic self-healing can occur repeatably but require a stimulus, for example, a mechanical force or elevated temperatures. To overcome this, materials utilizing other mechanisms like magnetic particles have been designed. Healing mechanisms have their own different limitations and characteristics, like hydrogen bonding can be impeded by aging or moisture but certain self-healing mechanisms are aided by water due to swelling of the polymer. There is a diverse range of self-healing materials based on different chemistries and interactions between the constituents in the composite.

In this review, we present an update and summary on the mechanics, strategies and potential applications of stretchable and self-healable conductors over the last couple of decades. We hope that this review will aid interested readers to better understand this area, and gain an appreciation of the importance of conductors and self-healing in stretchable electronics.

## Intrinsically conductive materials

A widely used strategy to realize stretchable conductors is to combine conductive fillers, which are generally rigid and brittle, with a stretchable elastomer [55], forming a blended composite block or thin film [56, 57]. The different properties of each component, mainly the electrical conductivity of a filler and stretchability of an elastomer, complement each other and generates synergy. There is a diverse range of conductive fillers, including carbon-based (e.g., CNT, carbon black (CB), graphene, graphite, carbon fiber), metal-based (e.g., silver (Ag), gold (Au), copper (Cu)) materials, and conductive polymers (e.g. poly(3,4-ethylenedioxythiophene): poly(styrene sulfonate) (PEDOT:PSS), polyaniline (PANi), polypyrrole (PPy)) [58]. There is also a wide range of geometrical shapes and sizes of the fillers (e.g. wire, flakes, particles, etc.). For the elastomer that fillers are dispersed into, PDMS, PU, Ecoflex, poly(styrene–butadiene–styrene) (SBS) are the most widely used materials [26]. The dispersed fillers in the polymeric matrix form conductive pathways where electrons can transport, rendering the composite electrically conductive. This percolation dependent conductivity is heavily influenced by the type and shape of the fillers. This determines the amount of junction contact resistances induced [59] and is also closely associated with the stretchability of the composite. Therefore, the selection of fillers and elastomer is of great importance.

**Fig. 2:**
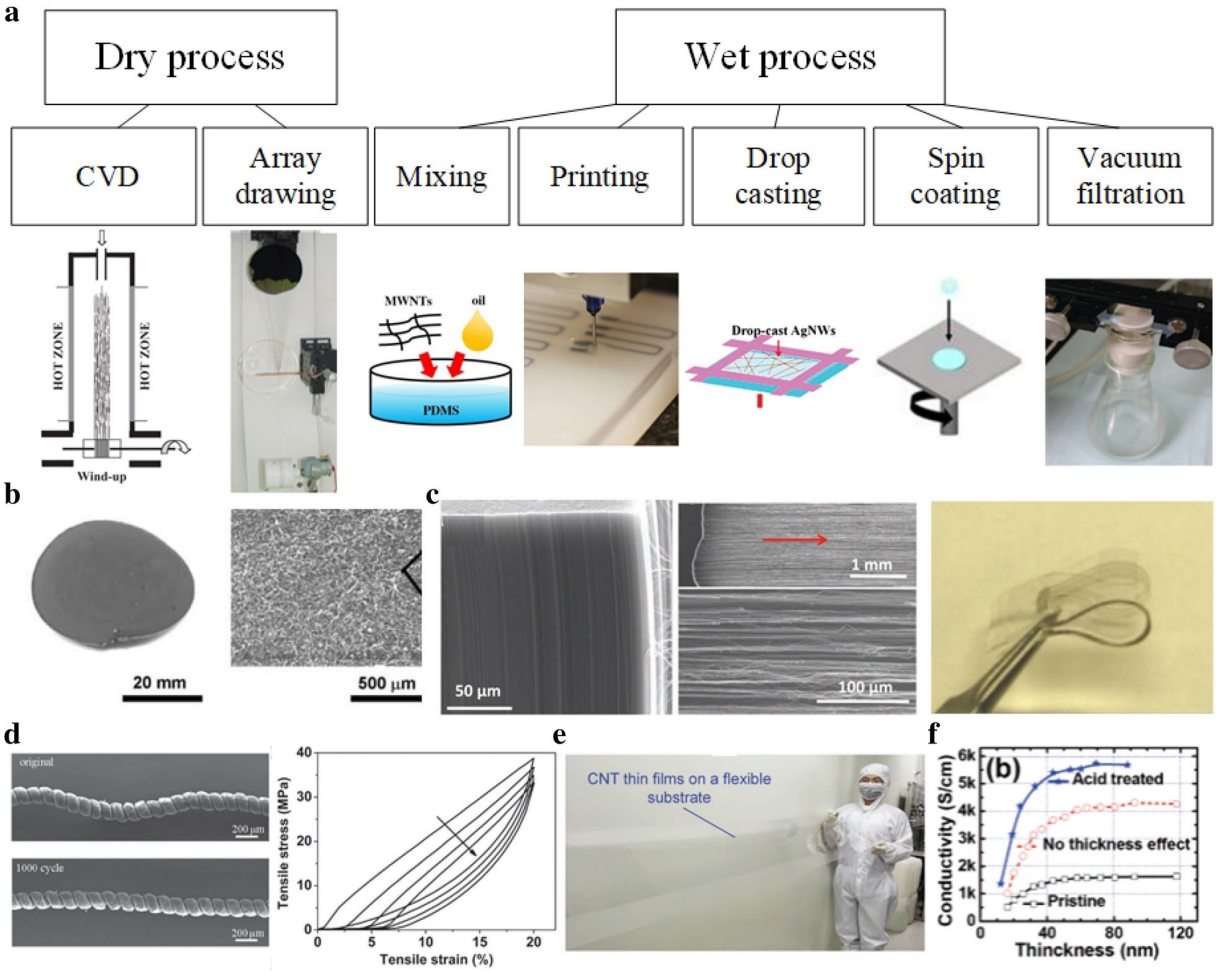
**a** Commonly used fabrication process of a stretchable conductor. Reproduced with permission [60]. Copyright 2004. American Association for the Advancement of Science. Reproduced with permission [61–63]. Copyright 2006, 2014, 2012. Wiley–VCH. Reproduced with permission [64]. Copyright 2018. IEEE. Reproduced with permission [65]. Copyright 2014. Royal Society of Chemistry. Reproduced with permission from [66]; published by MDPI, 2018. **b** The fabricated stretchable conductors made of 1D SWNTs. Reproduced with permission [67]. Copyright 2008. American Association for the Advancement of Science. **c** Superaligned CNT forest (left), CNT ribbons pulled out from CNT forest (middle), and optical image of folded CNT ribbon film embedded in PDMS. Reproduced with permission [68]. Copyright 2010. Wiley–VCH. **d** SEM image of the fabricated 3D CNT ropes and stress–strain curves for cycle 1st, 10th, 100th, and 1000th with a set strain of 20%. Reproduced with permission [69]. Copyright 2012. Wiley–VCH. **e** A transparent SWNTs thin film on a PET substrate with a meter-scale and their SEM image. Reproduced with permission [70]. Copyright 2018. Wiley–VCH. **f** Effect of acid treatment of CNT on [71]. Copyright 2007. American Chemical Society

Another important consideration would be to choose an appropriate fabrication method. Commonly used methods are represented in Fig. 2a. Dry process such as direct chemical vapor deposition (CVD) and array drawing can create a high-quality film, but they are accompanied by potential high costs. In contrast, although wet process is relatively inexpensive and simple, it is generally difficult to obtain a high-quality film. However, in terms of industrial application, solution-based processing has a great advantage. Solution-based processes such as mixing, drop-casting and spin-coating generally allow for low-temperature processing, large scale production, and deposition onto various substrates. These methods can reduce the cost and overcome limitations in substrate materials and size compared to conventional processes in semiconductor manufacturing. However, polymer surfactants or ligands that aid homogeneous dispersion of fillers are likely to interfere with charge transport which limits the conductivity. Meanwhile, inappropriate fabrication process can nullify advantages of the filler morphology. For example, usage of ultrasonication for homogeneous dispersion of fillers may shorten the length of the fillers, which negates the efficacy of the high aspect ratio fillers for stretchable conductors.

For these reasons, to realize the best performing stretchable conductor, diverse factors ought to be considered. The selection of materials for fillers and elastomer, and the fabrication process both determines the electrical and mechanical performances of the conductors. The list of stretchable conductors based on different conductive fillers is provided in Table 2. In the following section, we will discuss the strategies for high-performance stretchable conductors based on the materials.

**Table 2:** Summary of the performance of the stretchable conductor

Filler type	Substrate	Fabrication process	Maximum conductivity or sheet resistance	Maximum strain (%)	Refs.
CNT forest	PU	Drop-casting	1 S cm^−1^	1400	[27]
CNTs	PDMS	Spray coating	2200 S cm^−1^	150	[72]
CNTs	PDMS	Contact transfer	3316 S cm^−1^	285	[73]
CB	Styrene butadiene	Blending	40 S cm^−1^	200	[74]
Carbon grease	Ecoflex	3D printing	~0.05 S cm^−1^	700–800	[61]
Graphene	PDMS	Contact transfer	280 O sq^−1^	30	[75]
CNFs	Paraffin wax-polyolefin	Spray coating	10^1^–10^2^ O sq^−1^	500	[76]
AgNWs	PU	Blending	14,205 S cm^−1^	200	[77]
AgNWs	Ecoflex	Contact transfer	9–70 O sq^−1^	460	[62]
AgNWs	PDMS	Spray coating	~5 O sq^−1^	~40	[78]
Ag flakes/Ag NPs	Fluorine rubbers	Blending	6168 S cm^−1^	400	[79]
Cu–Ag NWs	Ecoflex/Dragon skin	Filtration and polymer infiltration	1220 S cm^−1^	350	[80]
AuNPs	PU	Layer by layer, vacuum-assisted flocculation	11,000 S cm^−1^	486	[81]
CuNWs	Poly(acrylate)	Drop-casting	2.6 O sq^−1^	17	[82]
CuNWs	PU	Drop-casting	8 O sq^−1^	85	[83]
CuNWs	Ecoflex	Vacuum filtration and laser annealing	18 O sq^−1^	250	[84]
PEDOT:PSS	PDMS	Spin-coating	550 S cm^−1^	188	[85]
PEDOT:PSS	SEBS	Blending	4100 S cm^−1^	800	[86]
PEDOT:PSS	PDMS	Spin coating	46 O sq^−1^	10	[87]
PANI	SEBS	Blending	15,000 S cm^−1^	300	[88]

### Carbon-based fillers

Usage of carbon materials as a filler has led to substantial progress for the development of intrinsically stretchable electrodes. CB, CNT, graphene, carbon nanofibers etc. are commonly used carbon-based fillers for stretchable conductors [76, 89–91]. Their main advantage lies in their outstanding electrical and mechanical properties as well as cost-effectiveness.

Among carbon-based conductive fillers, CNT may hold the most promise. This is attributed to the efficacy of high aspect ratio, a wide range of structures (e.g., ribbon, yarns), availability of mass-production, and cost-effectiveness [92]. 1D CNT may hold more promise than nanoparticles or flakes (e.g., CB) due to junction-less conductive pathways, which have high conductivity without compromising the stretchability. This is because high loading of fillers to have an efficient pathway [93, 94] may adversely affect the stretchability [95].

When commercially available CNTs are embedded into stretchable elastomer, homogeneous dispersion is necessary as homogeneous dispersion holds a key role in realizing the ultimate properties of the resulting composites and enables patterning by e.g. 3D printing. In practice, due to the high van der Waals forces, multi-walled nanotubes (MWNTs) normally exist in clusters and it is difficult to disperse them into the solvent. Dispersion homogeneity can be resolved with the help of a surfactant or by choosing an appropriate solvent [92, 93, 96].

The representative example of CNT/elastomer blended composite for highly stretchable CNT-based conductor was introduced by Sekitani et al. [67] (Fig. 2b). They fabricated a single-walled nanotube (SWNT) based elastic conductor by dispersing SWNTs/ionic liquid mixture into a fluorinated copolymer. The developed SWNT film exhibits a conductivity of 57 S cm^−1^ when unstrained. To increase the stretchability, they perforated the SWNTs film so it becomes a net-shaped film, which allowed them to stretch up to ~ 130%. Due to the high aspect ratio of the SWNTs (3 nm in diameter and > 1 mm in length), the conductivity is barely changed under uniaxial stretching by 38% or less, and the conductivity is still as high as 6 S cm^−1^ when stretched up to 134%.

Interestingly, CNTs in a specially assembled form such as CNT-ribbon or CNT-yarn have been developed due to their unique benefits: e.g. better mechanical strength or higher transmittance. CNT ribbons refer to the sheet of continuous CNTs that can continue up to meter scale with unidirectional alignment along the drawing direction (Fig. 2c). They are obtained simply by being pulled out from vertically grown super-aligned CNT forests (Fig. 2c) [68]. Such a drawn CNT film can be twisted or shrunk by alcohol, or other diverse spinning processes [60, 97–99] becoming a yarn [100]. These CNT yarns or fibers have their high strength to weight ratio (Fig. 2d) [60, 69, 101]. Some superior properties of ribbon and yarns over randomly distributed CNTs have been exploited to fabricate better stretchable conductors. Zhang et al. demonstrated the effect of the alignment of CNTs on the stability of the electric conductivity under strains (Fig. 2c). The CNT ribbon film, where CNT ribbon is embedded in PDMS, presents unchanged conductivity under repetitive stretching with strains up to 100%. The CNT alignment along their axial direction may increase the chance that they can remain in contact even after the CNTs slid in the axial direction due to stretching. The CNT ribbon based stretchable conductor, in addition to the electrical stability, also possesses desirable properties such as being ultrathin, lightweight and transparent, and have large-area processability. Meanwhile, CNT yarns or fiber which is another unique CNT assembly have been developed to overcome low stretchability of these yarns (the strain to failure generally occurs at a strain of less than 10%), maintaining high mechanical strength induced by strong van der Waals forces among nanotubes [98, 60]. An attempt to achieve high stretchability while maintaining their high toughness is to shape them into spring-like CNT rope (Fig. 2d). The resultant CNT rope can sustain tensile strains as high as 285%, which is 20 folds higher strain than that of the pure yarn, and toughness as high as 28.7 J g^−1^. The electrical conductivity of the rope is also high at ~ 440 S cm^−1^. Besides, under the moderate tensile strain of 20%, the spring-like rope exhibited a stable spring constant for 1000 loading cycles. Another approach, recently, CNT yarns with outstanding toughness (357.2 J g^−1^) has been developed by imitating muscle structures and poly(vinyl) alcohol infiltration [102]. The increased capability to absorb the stress allows them to withstand tensile strains up to 186%. This developed yarn exhibits electrical conductivity as high as 4.2 × 10^3^ S m^−1^, is lightweight and has the ability to be mass produced, thus it possesses good application potential.

Stretchable transparent conductors have become one of the indispensable components for many applications such as optoelectronics, thin-film solar cells and transistors. CNTs or graphene embedded elastomers can fulfill these demands (Fig. 2e). The small diameter of CNT (~ nm) effectively reduces light scattering compared to other 1D metal nanowires [73] and enables meter-scale transparent conductive film. The stretchable conductors with carbon-based fillers achieved so far have shown strong potential to meet the requirements of practical applications (e.g., touch screen that needs 80% transparency and 500 O sq^−1^). Liangbing et al. developed highly stretchable and transparent CNT films which were deposited onto 3 M VHB 4905 substrate [58]. This film shows good stretchability, accommodating strains up to 700%, in which the electrical resistance superlinearly increased with the strain. However, for industrial applications, high conductivity is usually required to operate integrated circuits [67]. A CNT film with increased transparency and conductivity was also reported by Lipomi group [72], where their developed CNT network electrode shows conductivities as high as 2200 S cm^−1^ at a strain of 150%, and transparency of more than 88%. In cyclic tests, the CNT bundles were the most morphologically optimized at the 1500th cycle, at which they exhibited the minimum resistance.

Despite the many advantages of CNTs, their relatively lower conductivity compared to other types of fillers present challenges. An increase in the conductivity of CNT composite is required without high loading of fillers which adversely affect stretchability and transparency. Approaches such as chemical doping and modification of functional groups of the CNT surfaces have been used to improve electrical conductivity (Fig. 2f) [71, 103]. Acid treatment is done by simply immersing CNTs into acids, resulting in reduction of sheet resistance. In an example by Geng et al., HNO_3_ treatment for 60 min reduces sheet resistance by a factor of 2.5 times with negligible change in transparency [71]. Meanwhile, Kim et al. carried out chemical doping with gold trichloride solution (AuCl_3_), demonstrating an increase in conductivity of the SWCNTs/PU web composite [103].

### Metal-based fillers

Despite the tremendous popularity of the CNTs for stretchable conductors, the relatively higher conductivity of metal (CNT based composite: < 100 S cm^−1^, metal based composite: > 100 S cm^−1^) led to substantial progress of metal-based stretchable conductors [104]. The representative metal fillers used for stretchable conductors are silver, gold, and Cu. Their shapes are diverse, ranging from nanoparticle (NP), nanowire (NW), nanosheets, to nanoflakes. The fabrication approach for stretchable conductors is similar to that of carbon-based, most of which are solution-based approaches including vacuum filtration, Meyer rod coating, and solution coating followed by transfer. They are generally achieved at relatively low temperature. Of the numerous metal-based nanofillers, the most popular metal used is silver due to its lower price than gold, and higher stability than copper. In addition, the relatively low fusing temperature of AgNWs which could lower the electrical resistance has made it more attractive. These advantages have pushed significant advances in Ag-based stretchable conductors.

**Fig. 3:**
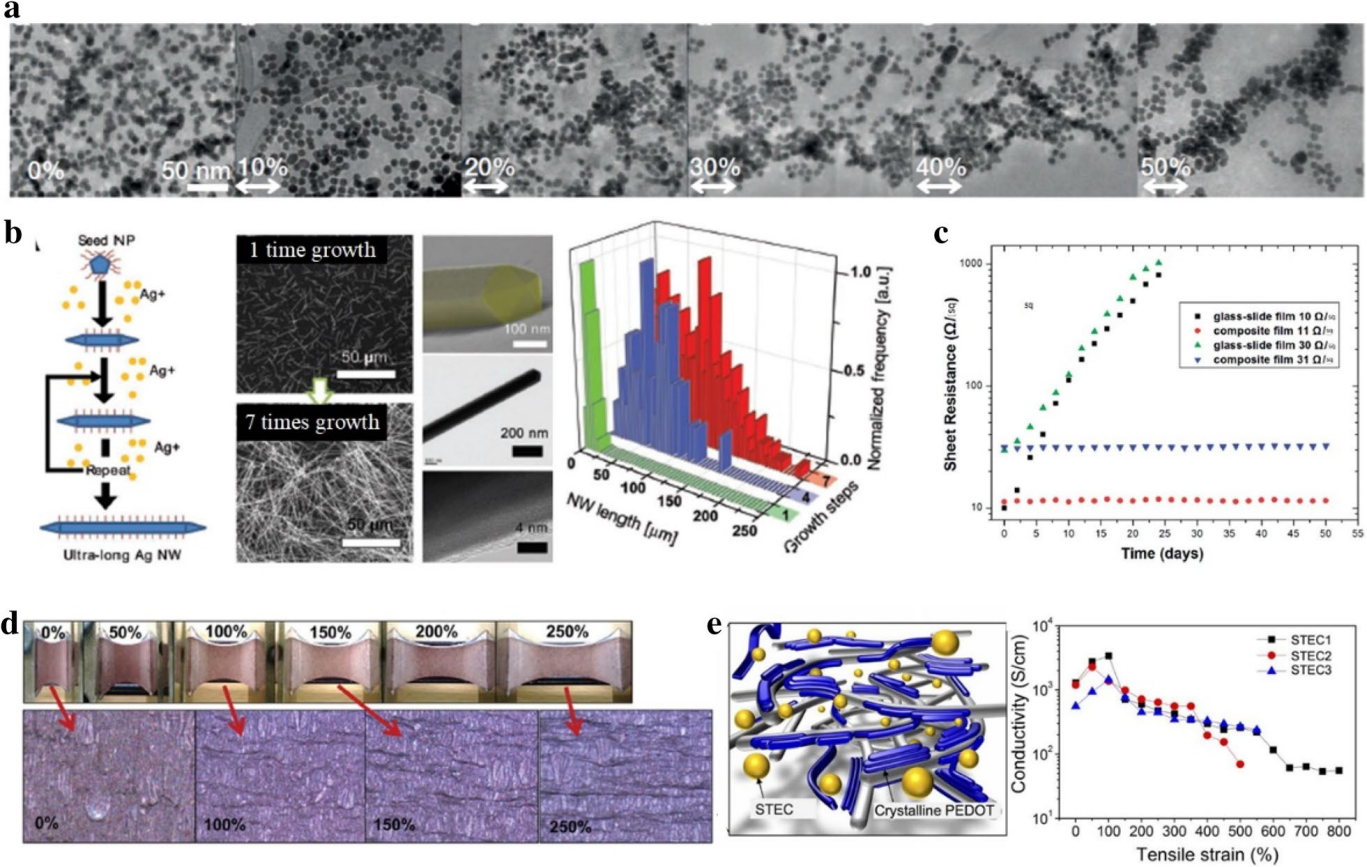
**a** Self-organizing behaviour of Ag nanoparticles under strain. Reproduced with permission [81]. Copyright 2013, Nature Publishing Group. **b** Synthesis process for very long Ag nanowires, and images (middle) and the NW length distribution graph (right) of lengthened Ag nanowires formed by the SMG method. Reproduced with permission [62]. Copyright 2012, Wiley–VCH. **c** Sheet resistance versus time of CuNWs based films to show the oxidative stability. Reproduced with permission [82]. Copyright 2014, Royal Society of Chemistry. **d** Laser-nanowelded stretchable CuNW conductor. Reproduced with permission [84]. Copyright 2014, Wiley–VCH **e** Schematic illustration of the PEDOT/STEC (left). Conductivity change under strain for PEDOT incorporated with three different enhancers for stretchability and electrical conductivity (STEC) (right). Reproduced with permission [86]. Copyright 2017. American Association for the Advancement of Science

Although 1D nanowires are generally regarded as the most effective fillers, gold nanoparticles exhibited outstanding performance as a highly stretchable and conductive material due to their unique behavior. By depositing AuNPs onto polyurethane through layer by layer (LBL) or vacuum-assisted flocculation (VAF) methods [81], films presented excellent electrical conductivity of 11,000 S cm^−1^ and 1800 S cm^−1^ under unstrained condition respectively, with corresponding maximum strains of 115% and 486%. When the respective films are stretched to a strain of 60%, the conductivity decreased to 3500 S cm^−1^ and 210 S cm^−1^, and when stretched to 110%, the conductivity decreased to 2400 S cm^−1^ and 94 S cm^−1^. The conductivity values still remain high even after strained, unlike other carbon-based conductors in spite of unfavorable low aspect ratio morphology. This is attributed to the self-assembly behavior of the AuNPs when stress is induced by stretching. As seen in Fig. 3a, as the tensile strain is applied, NPs are re-organized, aligned along the tensile direction. This re-organization and formation of conduction band facilitate the conductive pathway along the strain direction.

Apart from the special case of self-assembly behavior of NPs with effective mobility, 1D metal NWs normally show better performance as stretchable conductors. Going one step further, superior stretchability with relatively high electrical conductivity is realized with help of very long AgNWs synthesis called successive multistep growth (SMG) which enables an increase of the length to > 500 µm (Fig. 3b). The films with lengthened AgNWs accommodate strains above 460% while significant resistance change is not observed. The ductility that Ag intrinsically possesses, together with the more effective percolation network induced by long AgNWs under strain, produces a highly stretchable and conductive electrode.

Although AgNWs are the most extensively used fillers among conducting metal fillers, better cost-effectiveness (by 100 fold) and the higher abundance (by 1000 fold) of copper have attracted great interest in it as a promising conductive filler [105]. Nevertheless, a crucial drawback of copper that limits a wider use of copper fillers is susceptibility to oxidation which reduces the conductivity. In order to fully realize the advantages of copper (e.g., comparable electrical conductivity with silver), a diversity of strategies to avoid the oxidation problem without compromising electrical conductivity have been devised. They include coating with graphene oxide, nickel or chitosan [105, 106], or ligand [107], reduction of the oxide layer to metallic copper by acid solution [108], or embedment into elastomer [71, 82].

In addition, the welding process to reduce junction resistances of nanofillers would be hindered by the oxidized layer of copper fillers or give rise to oxidization of the fillers. Annealing processes under hydrogen or hydrazine [109, 110], or with the help of a protective layer (e.g., chitosan) have been carried out to overcome this oxidization problem. An example of embedment of CuNWs into the underlying substrate (e.g., elastomer) whereby oxidation is suppressed is represented in [82] (Fig. 3c). The polymer can effectively protect them from being oxidized by the surrounding air [82]. Compared to the CuNW film on glass, the films where CuNWs is partly embedded demonstrate excellent oxidative stability (Fig. 3c). This simple tactic allows for a transparent and stretchable conductor with chemical stability.

An example of nanowelding process with minimization of oxidation was introduced by Han and co-workers [84]. They conducted a plasmonic laser welding process to weld the nanowire junctions together. Such a process offers a reduction of resistance by minimizing the problem of oxidation incurred by conventional thermal annealing process. Furthermore, the prepared CuNW films show good stretchability (Fig. 3d).

Nanofillers play an important role to form conductive pathways that determine electrical conductance. Therefore, various post-treatment techniques to adapt to nanofillers can help facilitate the formation of conductive pathways. The as-synthesized nanofillers are normally capped with stabilizing ligands that hinder the conductive pathway. Processes of ligand washing to remove the thick ligand layer or ligand exchange to those with lower chain length can be carried out to lower contact resistance [104]. Also, soldering with additives [111] and welding [112] have also been performed to enable electron transfer at the junction.

### Conductive polymer based filler

Conducting polymer fillers in conjunction with carbon and metal fillers have been at the center of development of soft electronics. The main benefit of conducting polymer fillers is that they possess tunability of electrical and mechanical properties, through engineering the molecular structures or transforming into block copolymers that consist of the rigid electronic blocks and soft blocks [113]. Of all the conducting polymers, PPy, polyaniline (PANi), polyindole, polythiophene (PTh) are the most typical examples of conducting polymers studied [114, 115]. Unfortunately, their poor solubility may limit their wider use in applications.

On the other hand, a water-soluble conducting polymer, PEDOT:PSS has been developed, which comprises of positively charged PEDOT, and negatively charged and water-soluble PSS. Their solution processability, together with high conductivity, flexibility in tuning electrical and mechanical properties, transparency and commercial availability have made it an ideal material that can lead to significant innovation in stretchable conductors [113]. The additional advantage of solution processability is to enable large-scale production in soft electronics.

Although the high electrical conductivity of > 1000 S cm^−1^ can be achieved, coupled with good transparency of PEDOT:PSS (comparable to indium tin oxide, ITO), the fracture strain of PEDOT:PSS is as low as 5% and Young’s modulus is ~ 2 GPa [85]. The typical methods to improve mechanical compliance are the addition of plasticizers to lower the elastic modulus, examples of which include glycerol [116], Triton X-100 [117], and ionic liquids [118], or to form a polymeric blend with water-soluble polymers (e.g., poly(ethylene glycol) (PEG), poly(ethylene oxide) (PEO)).

The usage of architectures by buckling and kirigami methods discussed in the following section can be also considered. In an example (Fig. 3e) [86], an ionic liquid plasticizer allows PEDOT:PSS to dissolve in water and also plays the role of a dopant for PEDOT, improving its conductivity and elastic modulus. Excellent electrical and mechanical properties were obtained where the maximum conductivity is 3390 S cm^−1^ at the strain of 100%, and the fracture strain is 800% when supported on styrene ethylene butylene styrene (SEBS).

## Structural designs

Structural design is another important strategy in the fabrication of stretchable conductors. Certain structural designs can enable originally non-stretchable conductive materials to possess outstanding stretchability. Generally, different structural configurations can achieve different stretchabilities and remain relatively stable under applied deformation due to their specific geometric design. However, several challenges of applying this strategy have yet to be resolved by researchers in this field: (1) the fabrication methods of making stretchable electronic devices are complicated, (2) it often entails relatively high cost, (3) some of processing techniques are hard to control, making it difficult to achieve scalable manufacturing, (4) there usually are complications in the integration between stretchable interconnects and matrix.

**Fig. 4:**
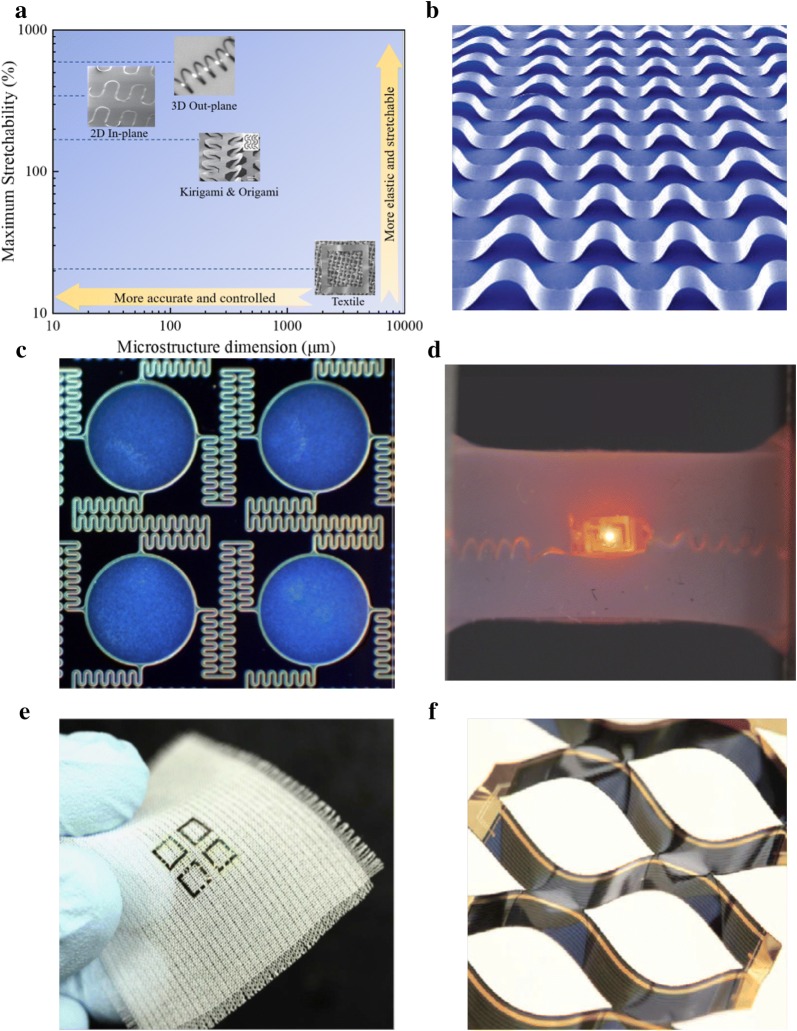
Structural design for stretchable conductors. **a** Comparison between 2D in-plane [119], 3D out-of-plane [120], textile [121], kirigami and origami designs [122] with aspects of maximum stretchability and microstructure dimension. **b** Buckling strategy and wrinkle conductor. Reproduced with permission [123]. Copyright 2006, Nature Publishing Group. **c** 2D in planed self-similar serpentine design. Reproduced with permission [124]. Copyright 2013, Elsevier B.V. **d** 3D out of planed helical design [125]. **e** Textile structural design. Reproduced with permission [121]. Copyright 2016, Nature Publishing Group. **f** Kirigami structural design. Reproduced with permission [126]. Copyright 2015, Nature Publishing Group

In this section, an intelligent pre-strain method to realize buckling design by Huang and Roger’s group is highlighted first as this method can be precisely controlled with an understanding of the buckling mechanism on a thin-film substrate, and thus it is considered as the most popular way in the area of making stretchable electronics. Then, different innovative structural designs developed by various research groups to realize stretchable functions are reported, including 2D in-plane, 3D out-of-plane, textile, kirigami and origami designs. Overall, these designs are summarized in Fig. 4a with the technique’s resolution and performance to give a brief comparison of these structural configuration designs.

### Buckling design

The buckling phenomenon was firstly applied by Huang and Roger’s groups in the stretchable electronics field [123]. It was found that a stiff ribbon which has poor stretchability can be attached to a pre-stretched elastomer surface at certain positions, and they become wrinkled after release, shown in Fig. 4b [127]. Earlier, it was also reported that the buckling phenomenon could be induced by thermal activation [128], however, this pre-stretch strategy is popular because it can be precisely controlled to have different ‘wavy’ structural configuration by using different pre-strain ratios [129], which were identified through theoretical equations [130] and finite element method (FEM) analysis [131]. Furthermore, it is clarified that pre-strain can cause both local buckling as well as global buckling phenomenon [132].

When attached to soft elastomers and stretched by pre-strain, rigid and brittle conductive materials are able to yield an outstanding mechanical elongation without breaking due to local buckling mechanism. However, global buckling may also happen during the process of pre-strain which is not very helpful with respect to stretchability. The boundary condition between these two buckling modes is computed analytically and proven by numerical simulation and experiment. By using different criteria of pre-strain ratio, layer thickness and adhesive bonding strength, different stages of buckling shapes would be formed, including non-buckling, local buckling or global bucking shape, and thus the choice controls the stretching performance [131].

Combined with the advance of conductive nanomaterial filled composites, this buckling strategy was widely exploited in stretchable conductors. Buckling mechanisms have been applied to materials such as CNTs [133], graphene [134], AgNWs [135] to fabricate stretchable conductors. The main advantage of these designs is the very small conductivity change when extended to the length of pre-strain [135]. It is worth mentioning that this strategy requires complicated manufacturing techniques such as thin film deposition, patterning, printing etc. Also, good integration of conductive material and substrate is required, achieved through methods like plasma treatment and addition of surfactant.

In addition to the pre-strain method, buckling shapes were also able to be achieved through stretch/release cycles. The buckling of AgNW/PDMS film [136] as well as laterally buckled CNT ribbons on PDMS [137] were reported using this method.

### 2D in-plane design

2D in-plane design represents a standard strategy for stretchable conductors. To realize this strategy, techniques such as relative lithographical patterning, coating and transfer printing, play an important role in adhering conductive materials to the elastomer surface. 2D in-plane design usually applies repeatable microstructures/patterns as the stretchable element. Each single unit is designed as a specific shape to provide its stretchability to the whole network. Fundamental stretchable patterns include several two-dimensional polygonous, serpentine filamentary networks [29, 138, 139] and other lattice shapes such as hierarchical triangular, diamond, hexagon and horseshoe [139, 119].

Many parameters e.g. type of metal material, line width and thickness of metal, lattice shape and corresponding aspect ratio will affect the whole structure’s mechanical performance. Moreover, to improve its stretchability and enhance its multi-staged mechanical behavior [140], hierarchical structures are further developed and investigated based on the original designs. In Fig. 4c, a self-similar serpentine interconnects design is shown. To serve as island-bridge in electronics, this two-order hierarchical configuration design demonstrates that its stretchability is improved by more than double compared to single serpentine design [124]. Among the lattice shapes mentioned above, fractal-inspired horseshoe designs provide the best stretchability performance supported by theoretical and FEM analysis [119].

By varying its arc angle, it can reach its optimum stretchability at an arc angle of 235 degrees. Thus, the high order fractal horseshoe microstructures have a substantially reduced elastic modulus as compared to traditional, first-order horseshoe designs. This design is advantageous for strain-limiting materials in bio-integrated applications due to its reduced levels of induced stresses.

### 3D out-of-plane design

Among all 3D out-of-plane configuration designs, helical structure is the most popular as it can be designed to achieve outstanding stretching ratios as well as higher robust mechanical performance compared to 2D structures [141, 142]. However, there is also the bonding issue in interfacial surfaces between conductive materials and elastomeric substrate due to its out-of-plane structural configuration.

Conventionally, 3D printing and mechanical guided method are two major processes to realize helical designs. 3D printing technology, including fused deposition modeling [143], ultraviolet(UV)-assisted [144] and solvent-cast 3D printing [145] can directly create spaced structures. However, only a few materials can be fabricated by the 3D printing approach and they tend to break easily while stretching.

Alternatively, Fig. 4d illustrates a mechanical guided approach to make a helical stretchable conductor, termed eHelix-Cu, and applied in a stretchable LED circuit as demonstration [125]. The micro-copper wire is wound around a barrel and then removed, becoming a spring-like structure. The wire was then under a surface treatment by using a silicone adhesive to provide a robust bonding between the copper wire and the silicone substrate. After embedding into a silicone rubber substrate, the helical interconnect and elastomer form a metal-rubber system which behaves as a highly conductive stretchable conductor that can be stretched with an outstanding strain ratio of 170%. Through understanding the interfacial mechanics of the metal and polymer, the embedded helical wire can reach very high strain ratio and then return completely with the support of polymer matrix, although the helical wire already undergoes plastic deformation. Yue et al. also showed that this method allows the conductivity to be unchanged during stretching cycles, and can remain so over thousands of stretching cycles.

The helical structures can also be generated from 2D serpentine shape by out-of-plane buckling. In this strategy, fiber-like serpentine ribbons are adhered onto the pre-stretched elastomeric substrate and fixed at some selected points, functioning as 2D conductors [146]. Upon releasing of the pre-strained substrate, ribbons transform from 2D wavy shape to 3D helical structure due to the local buckling mechanism mentioned previously. This geometric transforming method was studied quantitatively through specific simulation modeling [147]. Moreover, this strategy has been developed to form other innovative 3D structures, and it requires precise control of several parameters.

Other mechanically guided methods have also been introduced by researchers. A yarn-derived spring-like CNT rope was reported to have high stretchability by spinning technique [69], where a helical shape is formed by over-twisting of the yarn to form a structure consisting of self-assembled loops. Another way to make elastomer into a helical structure is by covering the screw with a straw, filling the gap with PDMS, and tearing off the curved PDMS. After that, CuNWs are transferred onto the surface of the helix structure of PDMS to increase its conductivity [120]. In addition, researchers also proposed a type of artificial muscle by using nylon 6,6 coil fibers to mimic the multi-level stress–strain behavior of the muscle [148]. Compared with the 3D printing method, the mechanical guided approach is easier to control and fabricate, but its microstructure dimension is relatively larger, at around 10 μm scale.

### Textile design

Textile design, considered as one of human’s oldest technologies, is also an interesting structural method of making stretchable conductors by weaving and knitting (traditional polymer composite reinforcement methods). Yarns and fabrics are environmental-friendly materials and are usually applied in clothes as the most fundamental ‘wearable device’ on the human body. Inspired by this, a method of combining electronic devices with clothes, using textile fabrication technology to create a brand-new stretchable and wearable device called ‘e-textile’ is shown in Fig. 4e [121]. The elastic performance of cotton knitted fabric composites was analyzed by FEM to show how different lengths of cotton knitted fabric specimens affect the stress–strain response [149]. Soft artificial muscles are also mimicked by electroactive polymers with textile processing to achieve mechanical stretchability and stability [150]. In this study, textile design enables scalable and rational fabrication of stretchable electronics. The same strategy is used for medical NiTi-braided devices [151] and fabric circuit boards [152].

### Kirigami and origami designs

Origami is a traditional art of paper folding technique which folds a piece of paper along the pre-defined crease patterns. A rigid origami could be understood as a mechanism with links and revolute joints. Plates are treated as links while hinges are regarded as revolute joints that connect two adjacent links. In recent decades, origami designs have been applied to engineering regime to alter traditional material behaviors [153]. When pre-defined crease patterns are applied on thick panels or engineering materials that allow bending or stretching, the structure will deform predictably. When stretching materials with crease patterns, the parallelogram faces (links) experience almost zero strains except at the deformable creases. At a small applied strain, the entire system has very low stiffness. As the applied strain increases, the folding creases straighten, and the parallelogram faces dominate the stretching such that the structure becomes stiff [154].

In summary, origami design creates potential ability of ‘foldable’ electronics. Recently, this design has been applied in flexible and foldable solar cells [155], thermoelectric nanogenerator [156], lithium-ion batteries [157] and photodetectors [158].

Kirigami is similar to origami but uses cuts instead of folds, which creates more flexibility compared to origami. Its mechanical behavior could be studied from its force–displacement curve as well as through FEM [159]. Figure 4f shows dynamic kirigami structures for integrated solar tracking device [126]. It is also reported that kirigami design has potential in applications of thin membrane devices [160] as well as stretchable energy storage devices [161]. The mechanical response of thin film material in which cracks or edges are designed in simple patterns is shown to have an initial stiff regime dominated by in-plane deformation and a highly stretchable regime governed by out-of-plane deformation [162]. Thus, the stretchability possessed by kirigami design is a result of a transition from the two-dimensional to three-dimensional deformation. It is shown that kirigami can usually achieve high stretchability (over 100%) for stretchable electronics [161] and that it could even reach up to 370% strain through certain patterned defect configurations [122].

## Self-healing materials

Self-healing can be classified as intrinsic or extrinsic: the former having self-healing functionalities inherent within the polymer, which can activate upon damage. Most intrinsic self-healing designs are based on reversible covalent bonds or supramolecular chemistry. External stimuli like mechanical pressure, electric or magnetic fields, heat and light may be required to initiate the self-healing. In extrinsic self-healing materials, a healing agent is separated from the polymer matrix, where damage will trigger the release of the agent. This healing process is usually autonomic.

Healing efficiency is defined as the ratio comparing a physical property of the healed material to its original value in pristine state. This could be ultimate tensile strength, fracture toughness or electrical conductivity, depending on the purpose of the material design.

### Extrinsic self-healing materials

**Fig. 5:**
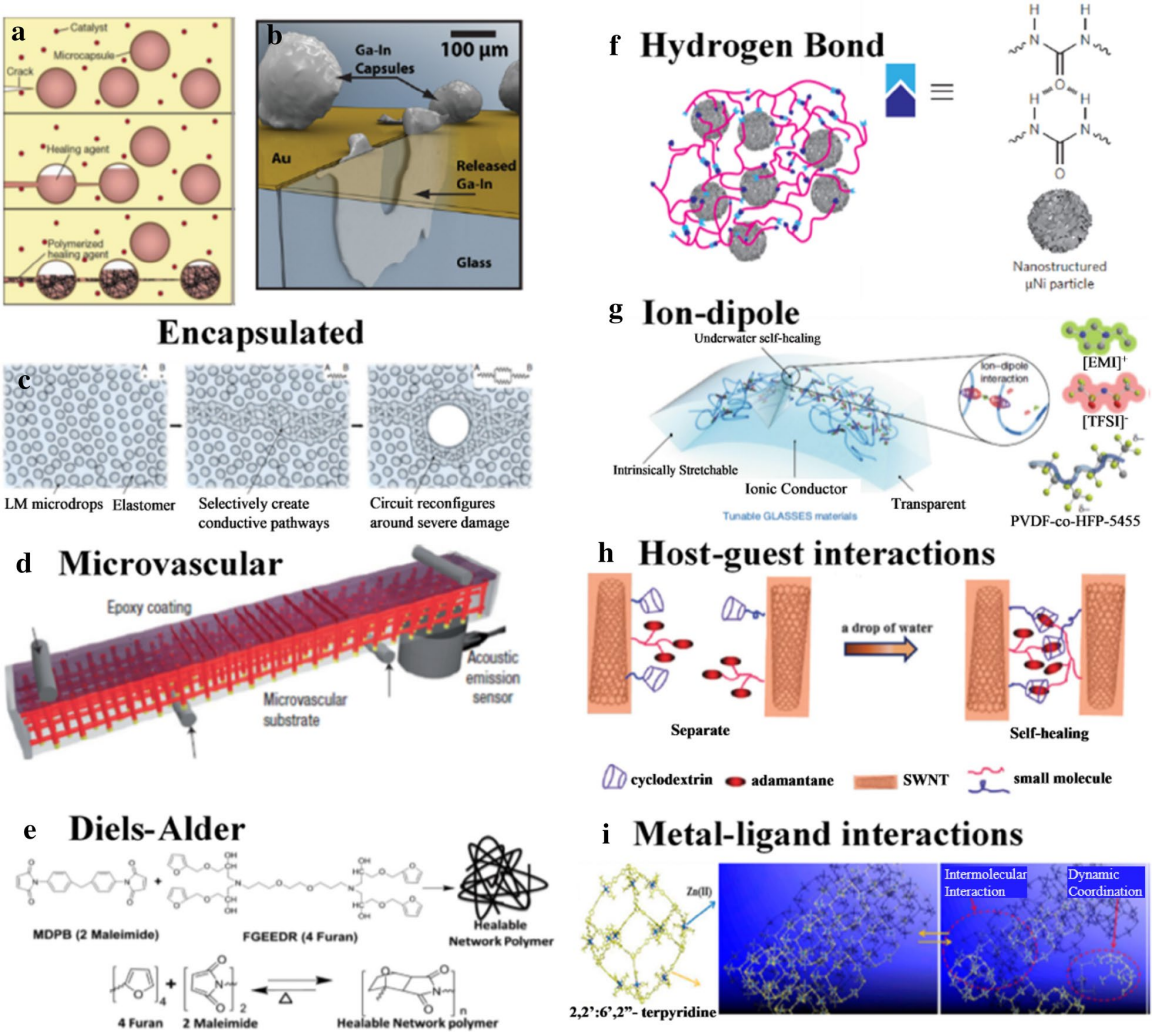
Self-healing Mechanisms. **a** Schematic of a crack propagating (top) releasing healing agent (middle) and repair of the crack (bottom). Reproduced in part with permission [163], Copyright 2001, Nature Publishing Group. **b** Schematic of released GaIn in crack plane. Reproduced in part with permission [165], Copyright 2012, Wiley–VCH. **c** Schematic of reconfiguration of encapsulated liquid metal for electrical conductivity. Reproduced in part with permission [166], Copyright 2018, Macmillan Publishers Limited. **d** Schematic of 3D microvascular system of substrate. Reproduced in part with permission [164], Copyright 2007, Nature Publishing Group **e** Diagram depicting a healable polymer using Diels–Alder chemistry. Reproduced in part with permission [167], Copyright 2013, Wiley–VCH. (f) Schematic of hydrogen bonding within a self-healing polymer. Reproduced in part with permission [168], Copyright 2012, Macmillan Publishers Limited. **g** Schematic of ion–dipole moments in aquatic self-healing polymer. Reproduced in part with permission [169], Copyright 2019, Springer Nature. **h** Schematic of self-healing via host–guest interactions. Reproduced in part with permission [170], Copyright 2015, The Royal Society of Chemistry. **i** Diagram showing Zn-tpy supramolecular structure and proposed mechanism of self-healing. Reproduced in part with permission [171], Copyright 2015, American Chemical Society

An early extrinsic self-healing polymer design utilizing encapsulated agents was reported by White et al. in 2001 [163]. The system was based on a healing agent, in this case, a liquid resin dicyclopentadiene (DCPD), encased in a microcapsule made of urea–formaldehyde. The microcapsules are then dispersed with Grubb’s catalyst in the polymer. When a crack propagates through the polymer, the microcapsules are ruptured, releasing the healing agent into the crack via capillary effect, where the catalyst initiates ring-opening metathesis polymerization (ROMP) upon contact (Fig. 5a). This method enables healing of the crack, recovering up to 75% of the original fracture toughness. However, this method is unable to heal multiple damages in the same region due to depletion of the healing agent. A 3-dimensional microvascular system filled with a healing agent (Fig. 5d) was later developed [164] to increase the number of healing events, but this was still limited by the availability of active catalyst after a few cycles.

Different systems have been developed since, some involving other healing agents such as reactive chemicals, suspension, solvents and liquid metals; with varying encapsulation strategies like single, double or all-in-one microcapsule. These are reported in detail by other reviews [172]. Design considerations include compatibility of the resin with polymer, low viscosity of the resin, toughness of the microcapsules, resin polymerization speed as well as stability to water, oxygen and temperature.

Although the previous examples were designed for recovering fracture toughness, there is also interest in recovering electrical conductivity, especially in the field of electronics and energy storage. Liquid metals are commonly used as they easily deform while maintaining conductivity and can flow to fill cracks. Encapsulated eutectic Gallium–Indium alloy (EGaIn) (Fig. 5b) has been shown to have excellent healing efficiency of more than 99% at a recovery time of less than 1 ms for a conductive circuit [165]. However, the design was on a non-stretchable substrate and did not include mechanical recovery.

In a more recent work [166], EGaIn droplets were dispersed in an elastomer (Fig. 5c), where the increase in resistance is less than 10% during stretching, tested up to 50% strain. Similarly, excellent electrical recovery was demonstrated as the liquid metal was able to form a conductive path around the region of damage, but there was no demonstration of mechanical recovery. In a separate stretchable wire design [173], a commercially available self-healing polymer (Reverlink, based on supramolecular chemistry) was molded into a wire with a microfluidic channel, where EGaIn was injected. The increase in resistance after the wire was cut and healed was around 3% but the maximum strain decreased from 150 to 60%.

The main advantage of using encapsulated healing agents is the ability to self-heal without external intervention and it has garnered great interest in fields ranging from building materials to electronics. However, in applications where the material undergoes many stretching and bending cycles, this method may not be suitable. The main drawback is the lack of repeatability due to depletion of the agent, especially when the strain concentrates at a certain region. Moreover, the dispersion of microcapsules or the incorporation of microvascular channels in the polymer matrix may disrupt the conductive network or reduce the mechanical strength of the polymer. Another challenge is the design and choice of healing agents which can recover both mechanical properties and electrical conductivity.

### Intrinsic self-healing materials

The second method for self-healing of stretchable conductors is to design polymer composites with reversible bonds, which can be covalent or supramolecular. The structure of these polymers is typically simpler than the first as there are no external healing agents or networks.

In the case of reversible covalent bonds, they are dynamic, able to break and form reversibly under equilibrium conditions, usually at elevated temperatures or with ultraviolet light. It is well-studied in the field of chemistry and polymer science, with numerous articles and reviews [174, 175]. However, as this achieves self-healing of the polymer but not the conductive network, there is a need for additional mechanisms to recover the electrical conductivity, e.g. good adhesion of the conductive particles to the polymer.

An example utilizes the Diels–Alder reaction [167], where the cycloaddition reaction between the furan and maleimide groups forms C–C bonds (Fig. 5e), which can break and reform upon heating to 110 °C. In this example, a conductive AgNW network was formed by drop-casting and the polymer was then coated on top. Upon inflicting a crack at the conducting surface, the conductive network reformed with the healing of the polymer substrate with an efficiency of 97%, but the conductivity decreased with subsequent healing cycles. A consideration for this method of self-healing is that the temperature required is relatively higher than ambient and may be detrimental to circuit components. It may be possible to lower the temperature at the cost of reducing the rate of healing.

Supramolecular bonds refer to non-covalent bonds between molecules; these include hydrogen bonds, ion–dipole interactions, metal–ligand coordination, π-π interactions, host–guest inclusion interactions and van der Waals forces. As their bond energies are lower than covalent bonds, they are typically able to reform at ambient temperatures. Many designs are based on simply applying a small mechanical pressure on the damaged parts to initiate self-healing.

A self-healing polymer based on hydrogen bonding between urea groups was developed by Leibler et al. [176]. A mixture of dimer and trimer fatty acids underwent a condensation reaction with diethylenetriamine which was subsequently reacted with urea, resulting in oligomers with hydrogen bonding. The hydrogen bonds form chains and cross-links between the oligomers, giving the polymer elastic properties. The low glass transition temperature allows the molecules to diffuse and achieve self-healing via the reformation of hydrogen bonds at any damaged surface. It was modified by Bao et al. [168] and nanostructured µNi particles were dispersed in the polymer to form a percolating conductive network. The surface of the µNi particles has a native oxide layer which can form hydrogen bonds with the oligomers, which aids in the good dispersion of the particles (Fig. 5f). When cut, the polymer can self-heal when a gentle pressure of 50 kPa is applied for 15 s. The healing efficiency for electrical conductivity was around 98%, whilst that for mechanical toughness was 41% after 10 min at ambient temperature, but more than 100% if heated to 50 °C.

However, self-healing utilizing hydrogen bonds may be inhibited by prolonged exposure to moisture, as water forms hydrogen bonds with the polymer at the damaged surface, reducing the ability of the polymer to self-heal. A recent work utilizes ion–dipole interactions to achieve self-healing of the polymer and resolves the problem of hydrogen bonding with water by appropriate choice of materials [169]. The hydrophobic supramolecular elastomer used, poly(vinylidene fluoride-co-hexafluoropropylene) p(VDF-HFP), possesses high dipole moments. An ionic liquid, 1-ethyl-3-methylimidazolium bis(trifluoromethylsulfonyl) imide ([EMI]^+^[TFSI]^−^) forms strong ion–dipole interactions with the polymer (Fig. 5g). Both the C-F groups in the polymer and the hydrophobic ionic liquid do not form strong hydrogen bonds with water. Thus, the material is capable of self-healing even when submerged in water. The ionic conductivity can be tuned by adjusting the percentage weight of the ionic liquid, up to 10^−3^ S cm^−1^.

Conversely, water can also be a stimulus for self-healing, and it is easily available. Although it has advantages in applications involving high humidity or aqueous mediums, the stability of the polymer needs to be addressed. Another concern is that electrical short-circuit may occur.

In an example using interactions between cyclodextrin (CD) host and adamantane (Ad) guest as the self-healing mechanism [170], water can enhance the recovery. CD was modified with pyrene, allowing it to be grafted onto SWNT via π-π interactions, forming β-CD-SWNT (Fig. 5h). These impart electrical conductivity and act as cross-linkers between the polyethylenimine oligomers which are modified with adamantane acetic acid (Ad-PEI). The conductivity was 0.15 S m^−1^, with almost 100% electrical recovery after cuts were made and the material pressed together. Mechanical strength recovery was 93% for the first cut and decreases gradually subsequently. Due to the tendency of PEI to absorb moisture, water can cause swelling of the polymer chains and aid diffusion and self-healing.

The conductive polymer PEDOT:PSS has been observed to self-heal in the presence of water [177]. A 40 µm gap was both electrically and mechanically healed in a strikingly fast time of 150 ms with a drop of de-ionized water. The mechanism is tentatively attributed to the swelling of the hydrophilic PSS^−^ chains. PEDOT: PSS films consists of grains of PEDOT^+^ core in a PSS^−^ shell, where electrostatic interactions hold the PEDOT and PSS chains together and hydrogen bonds between the sulfonate groups in PSS hold the grains together. The mobility of PSS^−^ chains is increased as hydrogen bonds between the sulfonate groups are broken in the presence of water, allowing them to propagate with PEDOT^+^ chains to the damaged area.

Conductive polymers can also be fabricated into hydrogels and aerogels. In a conductive self-healing hybrid gel developed [171], a PPy aerogel was first synthesized, then a supramolecular gel, G-Zn-tpy, was formed within the PPy matrix. The supramolecular gel consists of 2,2′:6′,2″-terpyridine (tpy) ligand and Zn (II) ions (Fig. 5i). Due to the metal–ligand coordination bond, as well as π–π interactions and hydrophobic interactions between the ligands, the supramolecular gel can self-assemble and reform around damage or cracks. The hybrid gel was tested to have a conductivity up to 12 S m^−1^, and when the sample was cut then pressed together, almost 100% electrical and mechanical healing efficiency after 1 min.

In an example using light as the external stimulus, conductive polymers were synthesized from azobenzene polymer, poly(disperse orange) (PDO 3), with AgNW [178, 179]. Self-healing was achieved by athermal photofluidization—where polarized light of 532 nm reduced the viscosity, enabling directional diffusion of the azobenzene to repair cracks. The AgNWs appear to diffuse with the polymer to restore electrical conductivity, this is attributed to good contact with the polymer.

## Applications

### Strain sensor

**Fig. 6:**
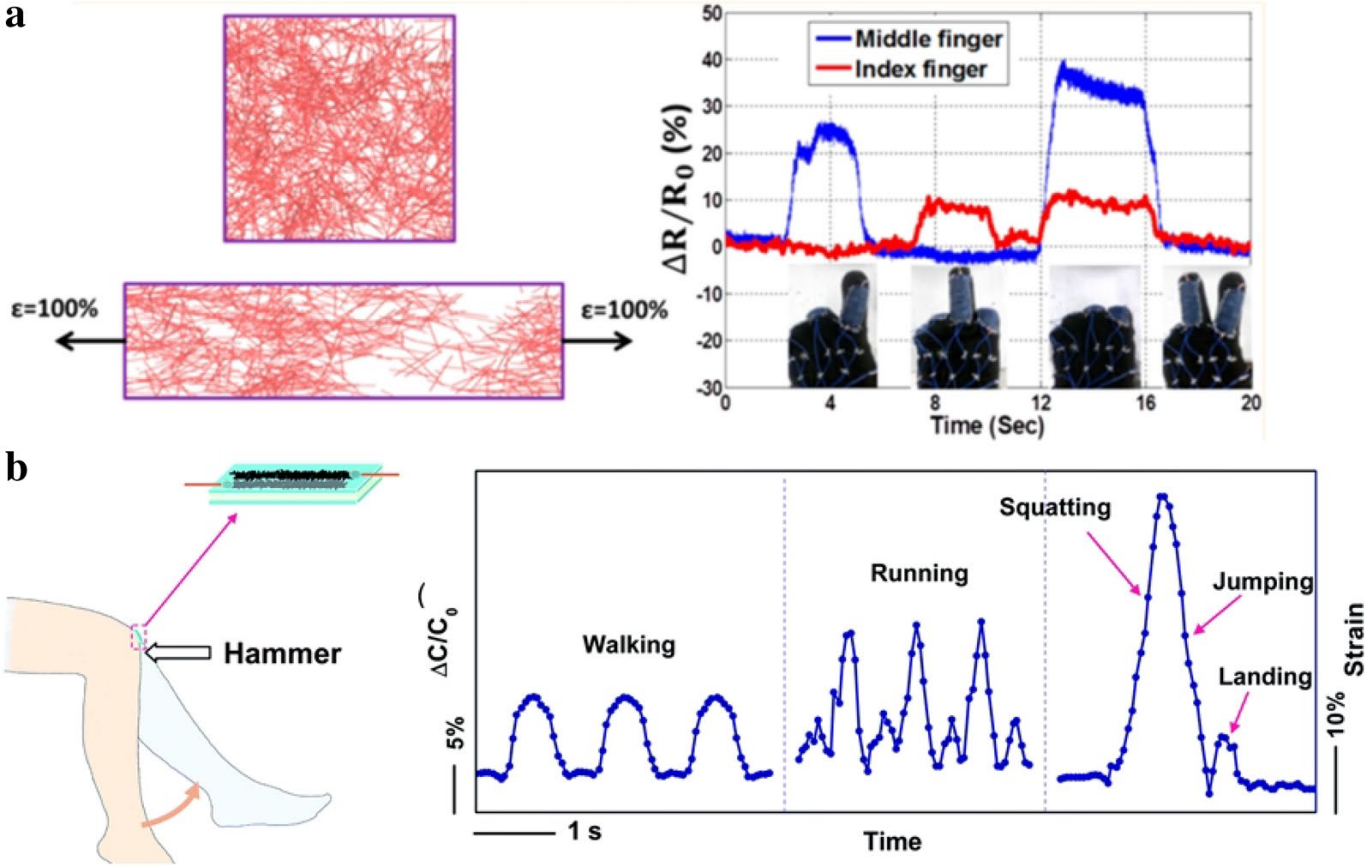
**a** Percolation dependent resistive type strain sensor. Disconnection of the AgNWs networks under strain (left), and relative resistance change under different finger motion (right). Reproduced with permission [59]. Copyright 2014, America Chemical society. **b** Relative capacitance change and strain as a function of time during diverse human motions of walking, running, squatting, and jumping by mounting strain sensor onto the knee. Reproduced with permission [44]. Copyright 2014, Royal Society of Chemistry

The tremendous progress of stretchable conductors has paved the way for realization of skin-mountable and wearable electronic devices. Strain sensors developed with stretchable conductors offer means to monitor the physical, chemical, and environmental status with minimum discomfort. The representative mechanisms in which strain sensors adopt are resistance change or capacitance change in response to the strain [180, 181]. One of the examples using resistance change in response to the strain was reported in Amjadi et al. (Fig. 6a). Stretching the AgNWs-elastomer causes slippage of the fillers and disconnection between neighboring conductive fillers and induces the loss of the percolation network, leading to an increase in resistivity (Fig. 6a). Due to the 1D conductive nanofillers that provide stable electromechanical property, this resistive type sensor showed high stretchability (70%), high gauge factor (2-14), and linearity.

One of the important parameters for good performance is linearity as it makes calibration relatively simple. Unlike many resistive type strain sensors which have poor linearity and hysteresis, capacitance type sensors generally exhibit good linearity. Capacitance type strain sensors are generally made of a deformable dielectric layer sandwiched by a pair of stretchable electrodes. Change in capacitance is encoded to strain. An example of capacitance type strain sensor with high sensitivity and good linearity is represented in [44] (Fig. 6b). The device detected diverse human motions well as its sensitivity is enough to detect target motion. An example that detects even subtle blood pulse signal and motions like blinking and clenching was introduced by Wang et al. [6], realized by graphene woven fabrics on PDMS. Monitoring of blood flow pulse is useful to check medical conditions like high blood pressure. Another performance parameter, increased sensitivity, which is usually quantified as a high gauge factor, is also important.

One strategy for this is to use conductive fillers with lower density networks whereby they facilitate the disconnection between filler junctions under strain and thus achieving higher gauge factors. Another approach to enhance sensitivity is to use treatment of nanofillers, which was conducted by Kim et al. [182]. The treatment of intense pulsed light irradiation onto composite where Ag flakes/Ag nanocrystals are embedded into PDMS leads to an increase in the gauge factor. In order to achieve high performance skin-mounted operation, apart from the stretchability, other properties such as lightness, biocompatibility, low power consumption are also desirable.

### Electrodes

Electrodes refer to electrical conductors that interface with a non-conducting part of the circuit. Examples of use include electrochemical cells, electrolysis and electrophysiology.

**Fig. 7:**
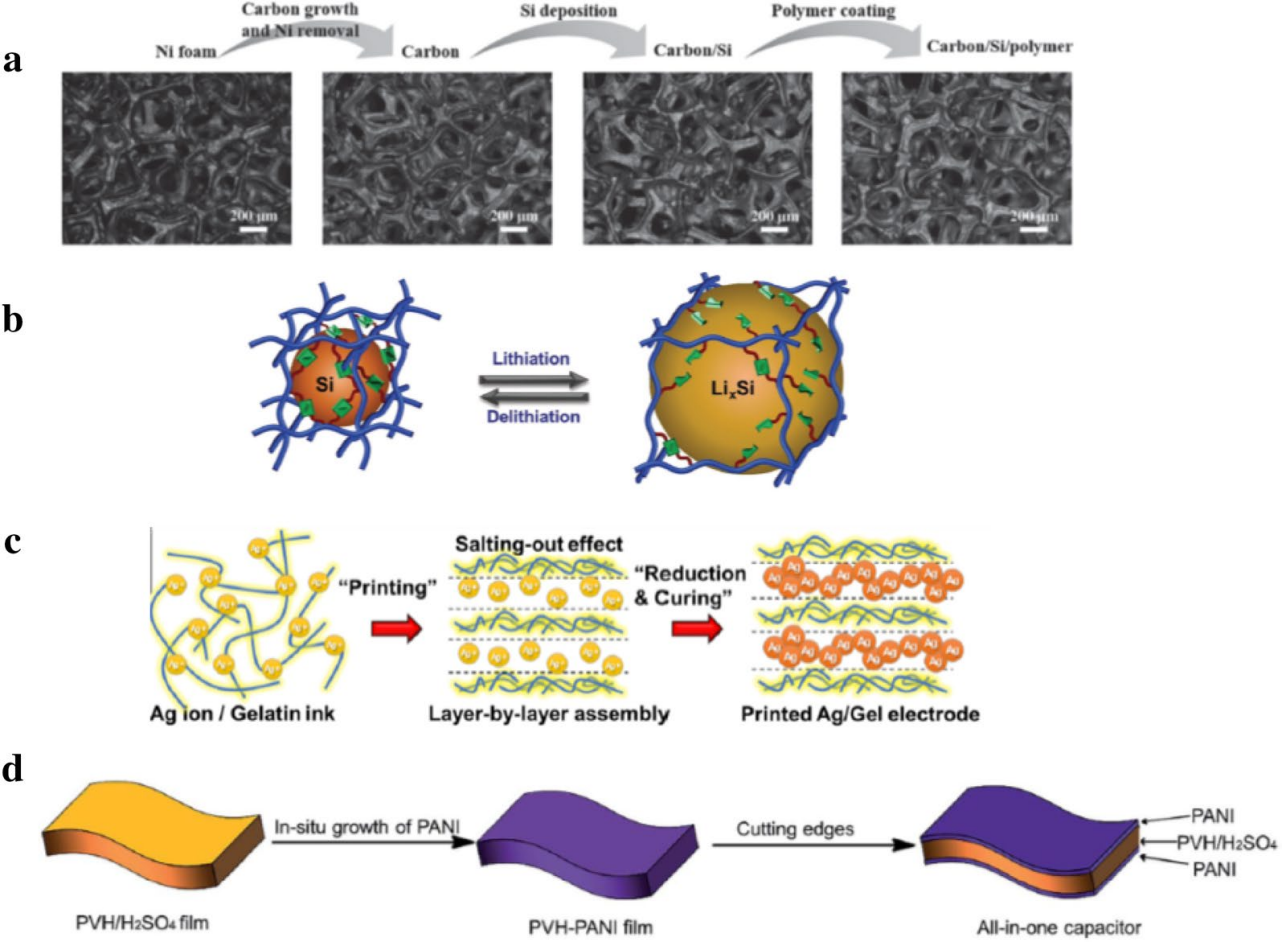
Illustrations of healable conductive electrodes. **a** Synthesis and microscope images of carbon/Si/polymer electrode. Reproduced in part with permission [183], Copyright 2016, Wiley–VCH. **b** Schematic illustrating volume change during lithiation/delithiation. Reproduced in part with permission [184], Copyright 2018, Wiley–VCH. **c** Schematic of self-layering mechanism of Ag nanosheets Reproduced in part with permission [185], Copyright 2018, American Chemical Society. **d** Schematic of capacitor fabrication. Reproduced in part with permission [186], Copyright 2018, The Royal Society of Chemistry

In lithium (Li)-ion batteries, although lithium metal anodes have a high theoretical specific capacity, there are safety issues because dendrites tend to form and cause a short circuit. Another material with high specific capacity is silicon, but the large volume change during lithiation and delithiation requires mechanisms to alleviate the stresses produced. A stretchable carbon/silicon anode for lithium-ion batteries was designed by coating with a self-healing polymer [183]. The fabrication process is illustrated in Fig. 7a. Graphitic carbon was grown by CVD on a nickel foam using vaporized hexane at ambient pressure. The nickel was subsequently removed, and a uniform layer of amorphous Si was deposited onto the 3D carbon skeletal structure using silane as a precursor at low pressure. The self-healing polymer was then drop-casted on the foam. It is a modified version of the hydrogen bonding based polymer in Sect. 4.2, but instead of reacting with urea, adipic acid was chosen.

Although the polymer had only partial healing when cut due to the breaking of non-reversible covalent bonds, stretching within 50% is unlikely to fully fracture the material and it can self-heal. The average strain beyond which the foam coated with 4× weight polymer is 46.2%, and 1000 loading/unloading cycles at 25% strain caused an increase in resistance of less than 400 Ω. A higher percentage of polymer increases stretchability but decreases specific capacity and efficiency due to its poor Li ion conductivity and increased diffusion length. The optimal was 4× weight polymer which showed only a slight decrease in specific capacity from 719 mAh g^−1^ to 584 mAh g^−1^ over 100 charge/discharge cycles.

In another work, poly(acrylic acid)- ureido-pyrimidinone (PAA-UPy) was chosen as the binder polymer due to the ability of UPy dimers to form quadruple hydrogen bonds [184]. Si NPs and conductive CB were dispersed in the polymer with weight ratio 6:2:2. The composite shows a high initial capacity of 4194 mAh g^−1^ and a capacity of 2638 mAh g^−1^ after 110 charge/discharge cycles. The adhesion force at cut surfaces was measured to be 36.2 mN m^−1^ after 1 min of contact, demonstrating self-healing. Combined with good adhesion with Si NPs and carbon black, crack formation was limited to widths of 0.3 µm after 110 cycles.

Recently, a printable conductive electrode was demonstrated by mixing gelatin with a Ag salt (silver trifluoroacetate) dissolved in ethanol and a reducing agent, dopamine, obtaining a gel ink [185]. A self-layering effect is shown in Fig. 7c, and Ag NP sheets were obtained after curing at 200-300 °C. The electrical conductivity could be tuned by varying the concentration of ethanol to control the surface morphology of the Ag NP layer. After 100,000 bending cycles of 2 mm radius (3.2% strain), the resistance of the electrode increased by less than 4%. Cracks produced by extreme bending could be healed by heating, and 100% electrical recovery was obtained at 150 °C for 5 min. This material was demonstrated as a printed flexible electrode for heating as well as for a micro-supercapacitor.

Typically, capacitors have interfaces between the electrodes and dielectric, which tend to delaminate during deformation, leading to interfacial contact issues. A self-healing capacitor was designed to overcome this problem by in situ integration of the electrode into a hydrogel electrolyte [186]. The hydrogel was synthesized from a co-polymer of vinylimidazole (VI) and hydroxypropyl acrylate (HPA), with ammonium persulfate (APS) and methylenebisacrylamide (MBAA) in an aqueous solution of H_2_SO_4_, forming PVH-H_2_SO_4_. It was then immersed in a solution of aniline (ANi) and APS added, where the ANi diffused into the hydrogel and was polymerized into PANi. The hydrogel was lastly cut to reveal the capacitor structure, shown in Fig. 7d. Self-healing was achieved due to the hydrogen bonding between the imidazole and hydroxy groups within the hydrogel, with additional cation–π and π–π interactions in the PANi layer electrode. After 10 cut/heal cycles at 25 °C for 20 min, the capacitance recovery was 96% while recovery of mechanical stress was 88.6%.

### Energy Harvester

Another important application of stretchable conductors is in energy harvesting devices. Development of energy harvesting devices is a step forward in the quest for an environmentally sustainable society. Emerging with stretchable electronics, energy harvesting devices have been the focus of researchers in the past decade. Prior to using stretchable conductors, most of the flexible energy harvesters can only operate under a low strain ratio (~ 0.1%) [187]. Thus, highly flexible and stretchable energy harvesting devices (over 15% strain ratio) are studied by applying the strategies of material development, structural configuration design and integration methodology. Based on their specific potential applications, mainly in wearable and stretchable electronics, energy harvesting devices have been developed, converting mechanical energy to electrical energy mainly in two ways: triboelectricity or piezoelectricity, which are also called nanogenerators [188, 189].

**Fig. 8:**
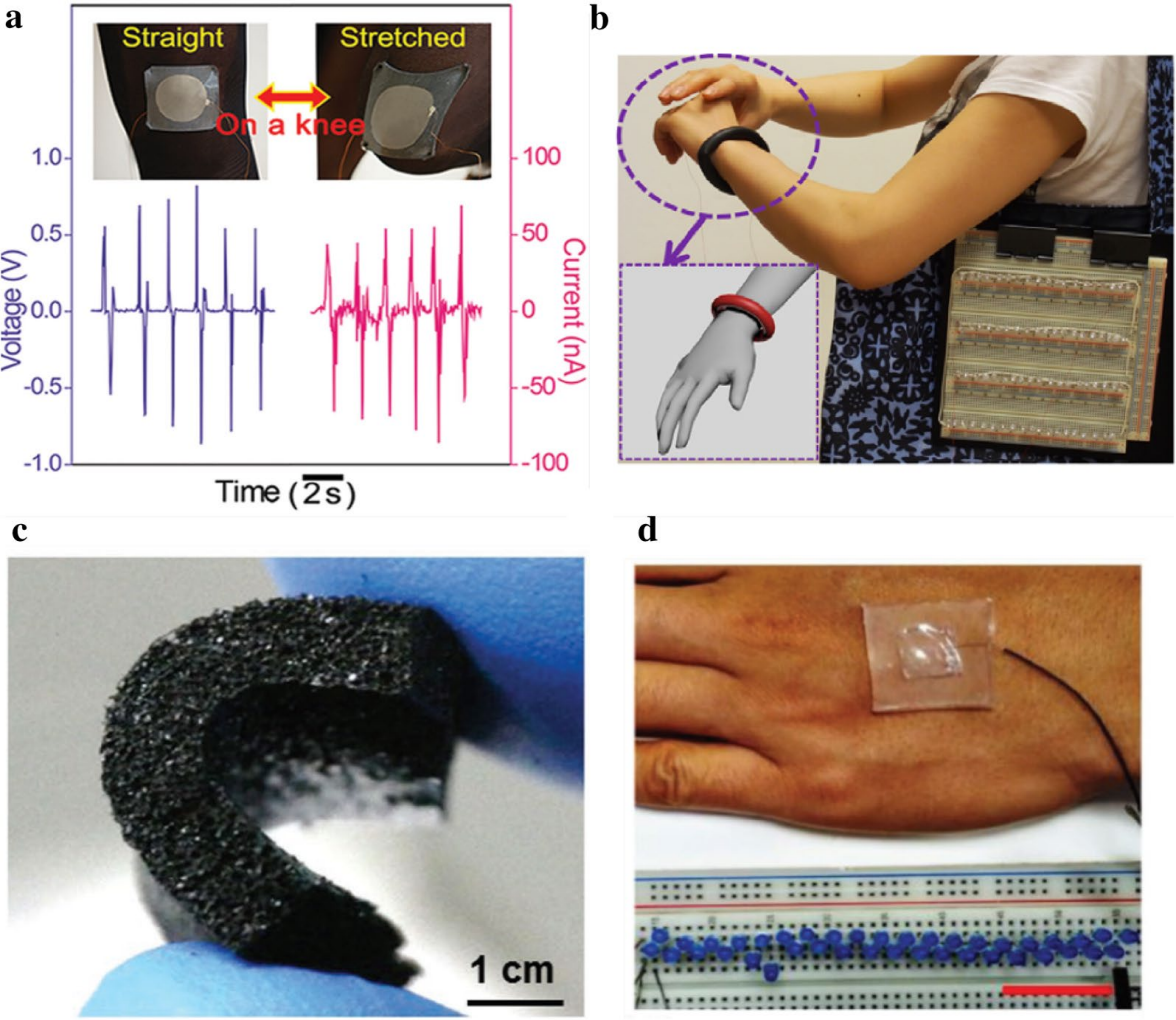
Stretchable and self-healable conductors for applications of energy harvesting. **a** A type of hyper-stretchable elastic-composite energy harvester. Reproduced with permission [190]. Copyright 2015, WILEY–VCH. **b** A highly shape-adaptive, stretchable design based on conductive liquid for energy harvesting and self-powered biomechanical monitoring. Reproduced with permission [191]. **c** Stretchable porous CNT-elastomer hybrid nanocomposite for harvesting mechanical energy. Reproduced with permission [192]. Copyright 2015, WILEY–VCH. **d** Highly transparent, stretchable, and self-healing Ionic-skin triboelectric nanogenerators for energy harvesting. Reproduced with permission [193]. Copyright 2017, WILEY–VCH

Recently, a hyper-stretchable elastic-composite generator (SEG) realized by very long Ag nanowires (VAgNWs) stretchable electrodes is reported to have high stretchability (around 200%) and about seven times higher power output than piezo-generators [190]. Figure 8a shows the generated voltage and current from the SEG on the stocking serving as a wearable device by bending and straightening the knee. A scalable approach for highly deformable and stretchable energy harvesters and self-powered sensors is also reported by fabricating a shape-adaptive triboelectric nanogenerator (saTENG) with conductive liquid contained in a polymer cover. It is claimed that this energy harvesting device can function as deformable and stretchable power sources and potentially applied in fields such as robotics and biomechanics. Figure 8b illustrates a saTENG looped around the arm of a subject to harvest energy from tapping motion and serve as a self-powered arm motion sensor.

A stretchable energy harvester can also be developed through combination with nanomaterials [194]. In this work, stretchable porous nanocomposite (PNC) based on a hybrid of a multiwalled CNTs network and PDMS matrix for harvesting energy from mechanical interactions is reported. A cross-section view of a bent PNC is illustrated in Fig. 8c. In addition, an innovative method of using an ionic conductor as the current collector in the energy harvester is demonstrated to enhance stretchability, transparency and self-healing properties [195]. The resulting ionic-skin TENG (IS-TENG) has a high transparency (92% transmittance), ultra-long uniaxial strain (700%) and good self-healing performance that can recover its performance after 300 times of complete bifurcation. Figure 8d shows the digital photo of this IS-TENG attached to the human palm as an energy harvester to power 40 LEDs.

## Conclusion and perspective

In this paper, we summarized the recent progress of stretchable and self-healable conductors including material development and geometric design strategies. We further described potential applications in wearable communication devices, biomedical engineering, healthcare monitoring, electrical artificial skin, soft robotics and transparent touch panels. The field of electronics is envisioned to shift increasingly from rigid electronic devices towards stretchable ones, where significant implementation advantages can be harnessed, especially when interfacing with soft human tissues.

However, there are still areas which require further development. So far, the conductivity of stretchable conductors in devices is still relatively low compared with traditional rigid electronics. In addition, the dimensions of stretchable electronic conductors might be still insufficient to reliably integrate with complex circuit designs. Furthermore, difficulties in integration between the electronic system and soft substrate still pose problems for the lifespan of stretchable devices. Moreover, generally low-conductivity of stretchable conductors could impede efficiently delivering power to various sensing devices. Currently, stretchable electronic devices are largely restricted to lab prototypes, and high-volume manufacturing presents challenges in scaling up such fabrication processes.

Overall, continuing research and development in this field is expected to produce a greater diversity of nanocomposite and/or composite materials for the design of stretchable conductors. Together with a deeper fundamental understanding of interfacial mechanics, new stretchable geometric designs, better mechanical and electrical performance in stretchable electronics can be realized. The use of manufacturing technologies will also increase the complexity and resolution of electronic devices, enabling large scale manufacturability. These developments can lead to the production of soft electronic devices that address the need for enhanced comfort, safety and functionalities in applications like healthcare and robotics. Continued advances in this area of soft electronics and advancing human capability to use these new form factors will be expected to bring enormous benefits to society and help to improve the quality of life for people. The future of electronic materials is an exciting and ever-evolving one, and it is certainly looking softer and brighter.

## Data Availability

Data sharing is not applicable to this article as no datasets were generated or analysed during the current study.

## Abbreviations

LED: light-emitting diode
PDMS: polydimethylsiloxane
PU: polyurethane
CNT: carbon nanotubes
CB: carbon black
PEDOT:PSS: poly(3,4-ethylenedioxythiophene): poly(styrene sulfonate)
PANi: polyaniline
PPy: polypyrrole
SBS: styrene–butadiene–styrene
CVD: chemical vapor deposition
MWNT: multi-walled nanotubes
SWNT: single-walled nanotube
NP: nanoparticle
NW: nanowire
LBL: layer by layer
VAF: vacuum-assisted flocculation
SMG: successive multistep growth
PTh: polythiophene
PEG: poly(ethylene glycol)
PEO: poly(ethylene oxide)
SEBS: styrene ethylene butylene styrene
FEM: finite element method
UV: ultraviolet
DCPD: dicyclopentadiene
ROMP: ring-opening metathesis polymerization
EGaIn: eutectic Gallium–Indium alloy
p(VDF-HFP): poly(vinylidene fluoride-co-hexafluoropropylene)
[EMI] + [TFSI]-: 1-ethyl-3-methylimidazolium bis(trifluoromethylsulfonyl) imide
CD: cyclodextrin
Ad: adamantane
Ad-PEI: adamantane acetic acid modified polyethylenimine
PAA-UPy: poly(acrylic acid)- ureido-pyrimidinone
APS: ammonium persulfate
PVH: co-polymer of vinylimidazole and hydroxypropyl acrylate
MBAA: methylenebisacrylamide
SEG: hyper-stretchable elastic-composite generator
saTENG: shape-adaptive triboelectric nanogenerator
PNC: porous nanocomposite
IS-TENG: ionic-skin triboelectric nanogenerator
PET: polyethylene terephthalate
SEM: scanning electron microscope
STEC: stretchability and electrical conductivity
